# Genome-Wide SNP discovery and genomic characterization in avocado (*Persea americana* Mill.)

**DOI:** 10.1038/s41598-019-56526-4

**Published:** 2019-12-27

**Authors:** Alicia Talavera, Aboozar Soorni, Aureliano Bombarely, Antonio J. Matas, Jose I. Hormaza

**Affiliations:** 1 Instituto de Hortofruticultura Subtropical y Mediterránea La Mayora (IHSM La Mayora -UMA-CSIC), 29751 Algarrobo-Costa, Málaga Spain; 2 Department of Biotechnology, College of Agriculture, University of Technology, Isfahan, 84156-83111 Iran; 3 School of Plant and Environmental Sciences, Virginia Tech, Blacksburg, VA USA; 4 Department of Biosciences Università degli Studi di Milano, Milan, Italy; 5 Departamento de Biología Vegetal, Universidad de Málaga, Málaga, Spain

**Keywords:** Plant breeding, Plant genetics

## Abstract

Modern crop breeding is based on the use of genetically and phenotypically diverse plant material and, consequently, a proper understanding of population structure and genetic diversity is essential for the effective development of breeding programs. An example is avocado, a woody perennial fruit crop native to Mesoamerica with an increasing popularity worldwide. Despite its commercial success, there are important gaps in the molecular tools available to support on-going avocado breeding programs. In order to fill this gap, in this study, an avocado ‘Hass’ draft assembly was developed and used as reference to study 71 avocado accessions which represent the three traditionally recognized avocado horticultural races or subspecies (Mexican, Guatemalan and West Indian). An average of 5.72 M reads per individual and a total of 7,108 single nucleotide polymorphism (SNP) markers were produced for the 71 accessions analyzed. These molecular markers were used in a study of genetic diversity and population structure. The results broadly separate the accessions studied according to their botanical race in four main groups: Mexican, Guatemalan, West Indian and an additional group of Guatemalan × Mexican hybrids. The high number of SNP markers developed in this study will be a useful genomic resource for the avocado community.

## Introduction

Avocado (*Persea americana* Mill.) is a subtropical evergreen tree native to Mesoamerica. Avocado belongs to the Lauraceae, a family in the order Laurales that, together with the orders Canellales, Piperales and Magnoliales, is included in the Magnoliid clade of early-divergent angiosperms^1^. This pantropical family has about 50 genera and 2500 to 3000 species. Besides avocado, only a few species in the family have economic importance and these include mainly spices [bay laurel (*Laurus nobilis* L.) and cinnamon (*Cinnamomum verum* J.Presl)], camphor (*C*. *camphora* (L.) J.Presl) and timber trees (*Nectandra* spp., *Ocotea* spp. and *Phoebe* spp.).

Traditionally, avocado genotypes have been classified in three horticultural races or subspecies mainly related to ecological preferences and botanical characteristics^2^. The Mexican and Guatemalan subspecies are adapted to highland areas in Central America (cold climates), being the Guatemalan race more susceptible to low temperatures. The West Indian subspecies is adapted to low-land areas in the same region (tropical climates).

Avocado market demand has increased exponentially in recent years and in 2017 avocado world production was close to 6 million tons. Most of the production is concentrated in a few countries (Mexico, Dominican Republic, Peru, Indonesia, Colombia, Brazil), Mexico being the largest producer with 34% of the total world production (more than 2 million tons)^3^. However, in spite of the increasing importance of this crop, there are important bottlenecks for efficient breeding and development of new avocado cultivars, due to the absence or poor availability of molecular resources and phenotypic data and to the limited genetic pool in breeding programs worldwide. Developing new high quality avocado cultivars is an urgent need in this crop since approximately 90% of the avocado production worldwide depends on a single cultivar, ‘Hass’, that originated as a chance seedling in California ninety years ago^4^.

Different types of genetic markers have been utilized in avocado for genotype fingerprinting, paternity analyses, diversity and phylogenetic studies, linkage map construction and screening for traits of interest. Initial works included minisatellites^5^, Variable Number of Tandem Repeats (VNTRs)^6^, Random Amplified Polymorphic DNA (RAPDs)^7^ and Restriction Fragment Length Polymorphism (RFLPs)^8,9^. More recently, Single Sequence Repeats (SSRs), which are codominant and highly polymorphic facilitating the study of intraspecific relations and diversity, have been specifically developed in avocado and used for fingerprinting and diversity analyses^10–19^. However, in spite of the inherent advantages of SSR markers, their frequency of distribution is not uniform over the genome and their use in association analyses is problematic^20^. Moreover, it is difficult to compare SSRs from different populations or systems, and the analyses are laborious and costly compared to new sequencing technologies (NGS)^21^. Indeed, Single Nucleotide Polymorphism (SNP) markers are becoming the marker of choice in crop genetic studies with different aims: linkage mapping, analysis of quantitative trait loci (QTL), association studies, marker-assisted selection (MAS) or genomic selection (GS)^22^. The advantages of SNPs include the large number of markers that can be generated at a reduced cost, the fact that they are the most frequent source of variation in eukaryotic genomes, their bi-allelic nature that offers accuracy in variant calling, their high reproducibility or their reduced cost that makes them accessible to most laboratories^23–25^. Those advantages are specially relevant in woody perennial crops since their application would significantly reduce time and cost of breeding programs.

Up to now, NGS applied to avocado research has been reduced to transcriptome analyses^26,27^ and the development of SNPs to characterize genetic diversity^28–30^. In addition, very recently, a first avocado nuclear genome sequence has been published^31^. In order to provide additional high quality SNPs for the avocado research community, in this work a collection of 71 avocado accessions representing the three classical botanical races were genotyped and characterized using newly developed SNP markers. Those markers were mapped to a draft genome of the most important avocado cultivar worldwide, ‘Hass’, in order to increase the quality of the markers developed.

## Results

### Development of an avocado draft genome for mapping the raw reads

A draft genome of the avocado ‘Hass’ variety was developed to assist with read mapping and SNP calling. The sequencing of ‘Hass’ DNA produced 487.54 million raw Illumina reads (73.13 Gb) and 487.21 million processed reads (72.15 Gb). The estimated haploid genome size for ‘Hass’ ranged from 1.33 Gb (17-mer) to 1.63 Gb (73-mer) with an estimated genomic heterozygosity ranging from 1.05% (73-mer) to 1.41% (17-mer). The stats are summarized in Table 1. The assembly size represents 77% of the estimated genome size (1.33 Gb). The total number of sequences indicates highly fragmented assemblies in which the average sequence size (0.54 Kb) and the L50 (0.68 Kb) are below the average plant gene length (e.g. 2.01 Kb for *Arabidopsis thaliana*) and, consequently, no gene structural annotation could be performed^32^.

**Table 1:** Summary of the *Persea americana* Mill. cv ‘Hass’ draft genome assembly.

Assembly Statistics	Contigs	Scaffolds
Total assembly size (Gb)	1.03	1.01
Total assembled sequences	2,096,006	1,852,224
Longest sequence length (Kb)	57.80	160.08
Average sequence length (Kb)	0.49	0.54
N50 index (sequences)	475,145	377,224
L50 length (Kb)	0.56	0.68

### GBS sequencing, mapping and variant calling

GBS (Genotyping-By-Sequencing) libraries for 71 avocado accessions (Table 2) were constructed and sequenced by Illumina HiSeq 2500 (1 × 100) and Illumina HISeq 4000 (2 × 150). The sequencing produced 405.93 million raw Illumina reads. After processing (see Methods), 345.37 million reads were obtained with differences among accessions in the number of reads (Supplementary Fig. S1

**Table 2:** List of the 71 Avocado accessions studied with SNPs in this work.

Accesions	SampleID	Code	Germplasm collection	Previous race assignment	Race assignment predicted from the results of this work
0028(Ardith)	2835	1	South Africa	GxM^85^	GxM
A0.25	A02554	2	South Africa	Unknown	GxM
A0.68	A06852	3	South Africa	Unknown	GxM
87.17.1	871728	4	South Africa	Unknown	GxM
1.14.2	114218	5	South Africa	Unknown	GxWI
Alcaraz	ALCA74	6	Spain	Unknown	GxM
Bacon	BACO39	7	South Africa	GxM^12^, M^11,41^ or G^40^	GxM
Bernecker	BERN18	8	USA	WI^86^	WI
Beta	BETA19	9	USA	GxWI^87^	GxWI
A0.57	A05720	10	South Africa	GxM^12^	GxM
Butler	BUTL16	11	USA	WI^85^	WI
C.A. Bueno	CABU95	12	Spain	Unknown	M
Catalina	CATA11	13	USA	WI^85^	WI
Choquette	CHOQ9	14	USA	GxWI^85^	GxWI
Cilfam	CILF46	15	South Africa	Unknown	GxM
Colin V-33	COLI31	16	South Africa	GxM^85^	GxM
Collinred B	COLL1	17	USA	GxWI^85^	GxWI
Collinson	COLL36	18	USA	GxWI^85^	GxWI
Dusa	DUSA33	19	Spain	GxM^12^	GxM
Edranol	EDRA63	20	South Africa	Hybrid^4^ or G^4^	GxM
Fuchsia	FUCH17	21	USA	WI^85^	GxMxWI
Fuerte	FUER16	22	South Africa	GxM^12^ or M^40^	GxM
G-6	G692	23	Spain	M^12^	MxWI
Gem	GEM77	24	Spain	GxM^12^ or G^41^	GxM
Gottfried	GOTT04	25	South Africa	M^88^	MxWI
Grace	GRAC26	26	South Africa	Unknown	GxM
Gwen	GWEN40	27	South Africa	GxM^85^ or G^40^	GxM
H287	H28757	28	South Africa	Unknown	GxM
Hansie	HANS05	29	South Africa	Unknown	M
Hass	HASS38	30	Spain	GxM^11,31^ or G^12^	GxM
Hass	HASS55	31	South Africa	GxM^11,31^ or G^12^	GxM
Iriet	IRIE34	32	Spain	GxM^11^	GxM
A0.67	A06729	33	South Africa	Unknown	GxM
Lamb Hass	LAHA24	34	South Africa	GxM^11,12^	GxM
La Piscina	LAPI93	35	Spain	Unknown	M
Largo	LARG24	36	USA	WI^85^	GxWI
Linda	LIND50	37	South Africa	G^85^	G
Lisa	LISA23	38	USA	MxWI^85^	GxMxWI
Lyon	LYON25	39	South Africa	Hybrid^41^ or G^85^	GxM
Maluma	MALU85	40	Spain	GxM^4^	GxM
Melendez 2	MELE12	41	USA	GxWI^85^	GxWI
Mike	MIKE30	42	South Africa	Unknown	G
Monroe	MONR10	43	USA	MxWI^85^ or GxWI^85^	GxWI
Mrs Tooley	MRTO08	44	South Africa	Unknown	GxMxWI
Murrieta Green	MUGR27	45	South Africa	G^41^	G
Nabal	NABA21	46	South Africa	G^85^	G
Negra de la Cruz	NECR31	47	South Africa	M^89^	GxM
Nimlioh	NIML09	48	South Africa	G^85^	G
Nn10	NN1068	49	South Africa	G^41^	GxM
NN63	NN6310	50	South Africa	G^41^	GxM
Pinkerton	PINK45	51	South Africa	GxM^12^ or G^40^	GxM
Pollock	POLL6	52	USA	WI^85^	WI
Reed	REED89	53	Spain	G^41^	GxM
Regal	REGA11	54	South Africa	Unknown	GxM
Rincon	RINC12	55	South Africa	Unknown	GxM
RR-86	RR8691	56	Spain	Unknown	GxMxWI
Rustenburg Round	RURO36	57	South Africa	Unknown	GxMxWI
Russell	RUSS22	58	USA	WI^85^	WI
Ryan	RYAN13	59	South Africa	GxM^85^	GxM
Semil 43	SEMI14	60	USA	GxWI^86^	GxWI
Shepard	SHEP42	61	South Africa	G^41^	GxM
Teague	TEAG60	62	South Africa	M^41,85^	GxM
Telez	TELE66	63	South Africa	Unknown	MxWI
Thomas	THOM90	64	South Africa	M^12^	MxWI
Toro Canyon	TOCA96	65	South Africa	M^12^ or GxM^16^	GxM
Trapp	TRAP2	66	USA	WI^85^	WI
TX531	TX5344	67	South Africa	Hybrid^41^ or G^85^	GxM
Vero Beach n° 1	VERO4	68	USA	MxWI^85^	MxWI
Waldin	WALD28	69	USA	WI^85^	WI
Wester	WEST5	70	USA	WI^85^	WI
Yon	YON3	71	USA	GxWI^85^	GxWI

The race codes stand for: G = Guatemalan; M = Mexican; WI = West Indian. Interracial hybrids are indicated with a cross.

). A higher number of processed reads is often associated to a higher number of mapped reads to each of the GBS locations. These reads of the individual genotypes were mapped onto the reference genome to retain only mapped reads to a unique localization in the genome. Such uniquely mapped reads represented approximately 80% of the total. Finally, 1,070,902 variants were detected. Of those, 945,064 were SNPs, 22,321 were InDels, 69,500 were MNPs (multi-nucleotide polymorphisms) and 6,604 were complex (as combination of the previous types).

### SNP development

After filtering (see Methods), 7,108 SNPs with no missing data, of which 19.45% were private (Supplementary Table S1), were detected for the 71 accessions (Table 2). The SNPs were categorized according to nucleotide substitutions: 61.04% were transitions [C/T (2195) or A/G (2144)] and 38.96% transversions [A/C (778), C/G (646), A/T (666), G/T (679)]. The transition/transversion ratio was 1.57, similar to the results reported in other species^33–35^. The mean of observed heterozygosity was 0.16 whereas the mean of expected heterozygosity was 0.17 and the average frequency of minor alleles was 0.11, although, for the samples studied, the population was not in Hardy-Weinberg equilibrium. This last result was expected taking into account that the material studied does not represent a randomly obtained population.

### Diversity and population structure using filtered SNPs

**Figure 1:**
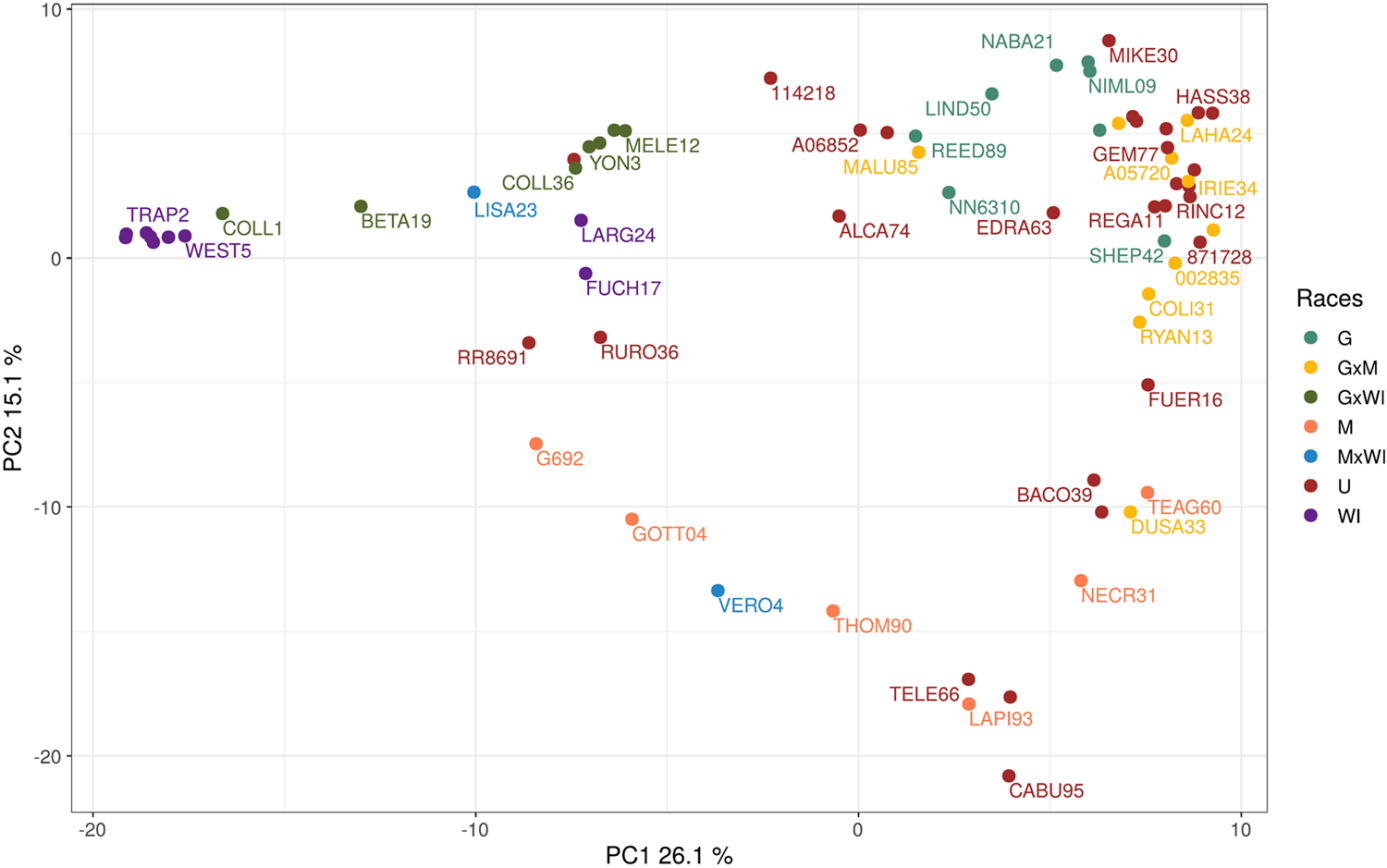
Principal component analysis (PCA) of 71 avocado accessions with 7108 SNPs using the R software version 3.5.1 with the package ggplot2 version 3^74^. Each genotype is represented with its sampleID (Table 2). The colors explain the race of the accessions according to the literature: turquoise green: G, yellow: GxM, dark green: GxWI, orange: M, red: U, orange: M, blue: MxWI, and purple: WI. (G: Guatemalan, M: Mexican, WI: West Indian and U: Unknown).

Distinct relationships among accessions were obtained with different analyses of the filtered SNPs. A first approximation to study genetic structure was obtained using principal component analysis (PCA) for the complete set of biallelic SNPs (Fig. 1). The first two components explained more than 40% of the variation (26.1% and 15.1%). Three differentiated groups that correspond with the three different horticultural races were observed. As expected, interracial hybrid accessions could be observed between the three main groups.

**Figure 2:**
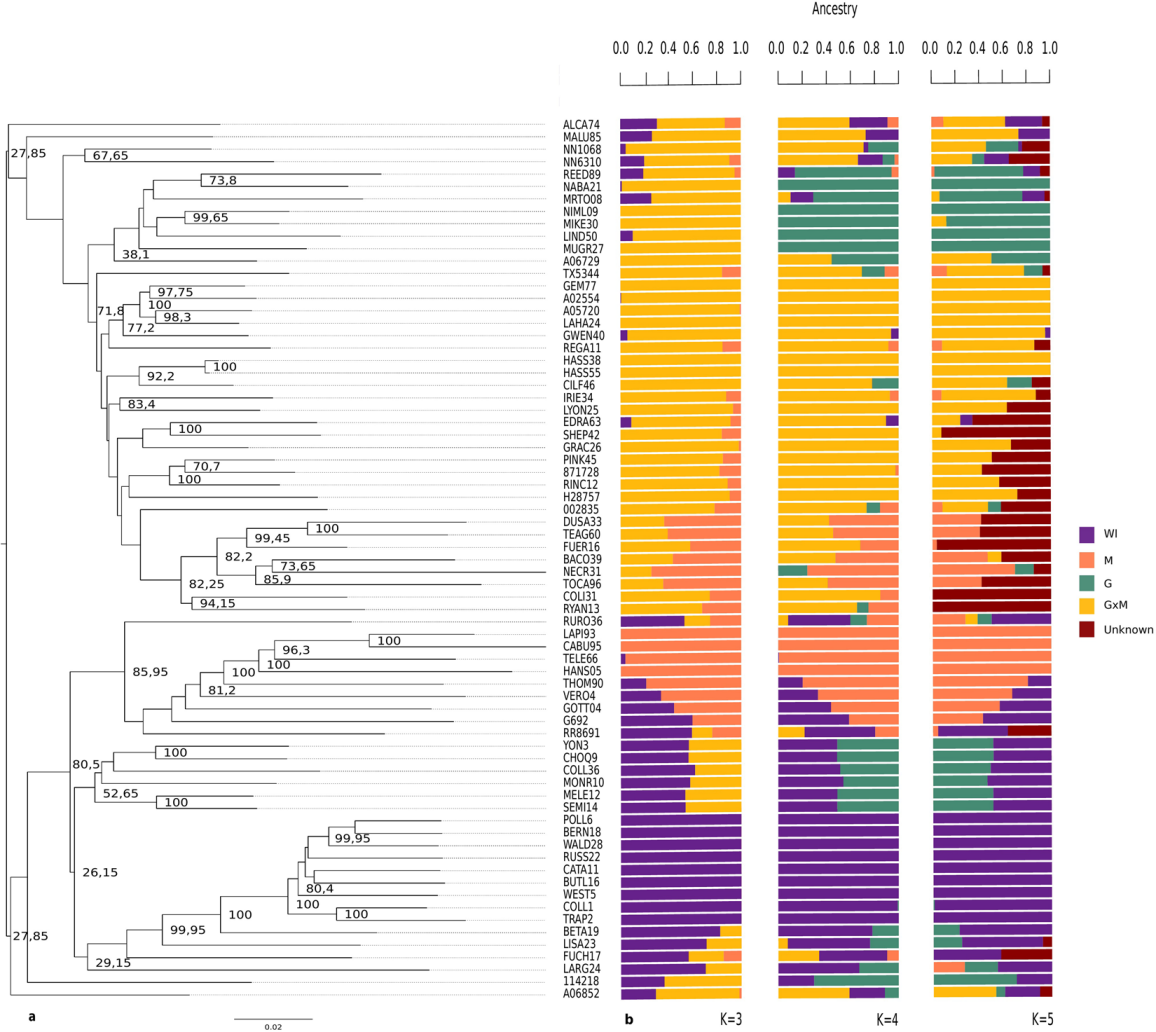
(**a**) Dendrogram based on Neighbour Joining (NJ) plotted using Figtree^78^ showing genetic relationships among 71 avocado accessions. Node labels represent bootstrap values (only values cited in the manuscript and values >70% are shown) out of 2000 bootstrap replicates. (**b**) Barplots describing the population stratification of the most probable number of clusters K = 4, followed by K = 3 and K = 5 were estimated with the ADMIXTURE software^37^. At K = 4, the avocado races were shown with different colors: orange: M; green: G; yellow: GxM hybrids; purple: WI; maroon: unknown. (G: Guatemalan, M: Mexican, WI: West Indian).

Prevosti’s distance^36^ was used to evaluate the genetic structure as a second approximation. This distance determines the fraction of different sites between samples. It was plotted as a dendrogram based on Neighbor Joining (NJ) showing the relationships between genotypes (Fig. 2a). Two main clusters weakly supported by bootstrap values (27.8) were revealed in the dendrogram. One of the clusters was composed of a big strongly supported subgroup (71.8) which included mainly Guatemalan x Mexican (GxM) hybrid genotypes (‘Pinkerton’, ‘Lyon’, ‘Iriet’, ‘Gem’, ‘Hass’, ‘Lamb Hass’, among others), a few genotypes categorized as Mexican (‘Teague’, ‘Negra de la Cruz’), as well as genotypes considered as Guatemalan (‘Shepard’), and a genotype of unknown race (‘TX531’). Another subgroup (bootstrap value of 38.1) included mainly accessions considered as Guatemalan (‘Reed’, ‘Nabal’, ‘Nimlioh’, ‘Linda’, ‘Murrieta Green’) and it was close to genotypes of unknown race (‘A0.67’, ‘Mike’,‘Mrs Tooley’). Moreover, the other two genotypes that are reported as Guatemalan (‘NN10’, ‘NN63’) form a strongly supported cluster (67.6), whereas ‘Maluma’ and ‘Alcaraz’ appear isolated of these subgroups.

The second cluster was formed by two genotypes of unknown origin (‘A0.68’ and ‘1.14.2’) and a strongly supported group (bootstrap value of 80.5) composed of two subgroups. One of them (well supported with a bootstrap value of 85.9), contained genotypes considered as Mexican (‘G-6’, ‘Thomas’, ‘Gottfried’), a MxWI hybrid (‘Vero Beach No. 1’), as well as genotypes of unknown race (‘RR-86’, ‘Telez’, ‘Rustenburg Round’, ‘C.A. Bueno’ and ‘Hansie’). The other subgroup was weakly supported (bootstrap value of 26.1) and was composed of two subgroups. One of them (29.1 bootstrap value), contained mostly West Indian genotypes (‘Pollock’, ‘Bernecker’, ‘Waldin’, ‘Russel’, ‘Catalina’, ‘Butler’, ‘Wester’, ‘Trapp’, ‘Fuchsia’,‘Largo’), together with some Guatemalan × West Indian (GxWI) (‘Beta’, ‘Collinred B’) or Mexican x West Indian (MxWI) (‘Lisa’) hybrids. The other subgroup was also weakly supported (52.6), and was represented by GxWI hybrids (‘Yon’, ‘Choquette’, ‘Collinson’, ‘Melendez 2’ and ‘Semil 43’) and a MxWI hybrid (‘Monroe’).

An admixture analysis using the ADMIXTURE software^37^ was performed after the PCA analysis. The most favorable number of clusters was 4, followed by 3 and 5 although the differences among the number of populations were small with a cross-validation error between 0.28 and 0.29. At K = 4, the division between genotypes reported as Mexican, West Indian and Guatemalan was evident. Furthermore, a separated cluster was formed with the GxM hybrid genotypes (Fig. 2b). In order to have a broader view of the genetic structure of the populations, the STRUCTURE software^38^ and STRUCTURE HARVESTER^39^ were also implemented. In agreement with the ADMIXTURE results, K = 4 was revealed as the most probable number of clusters (Supplementary Figs. S2 and S3b) but, in this case, accessions considered as Guatemalan and as GxM hybrids were not clearly differentiated.

**Figure 3:**
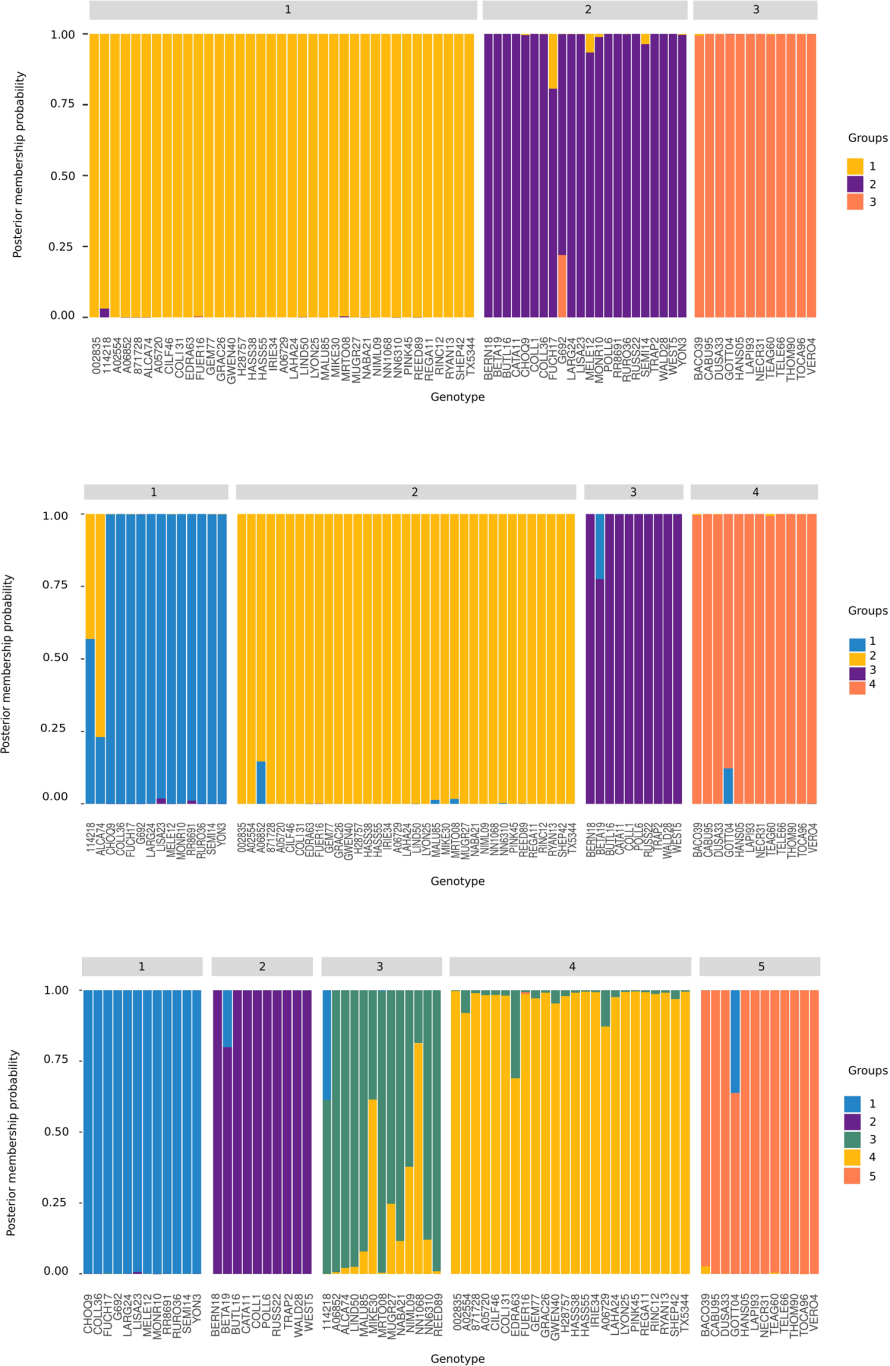
Discriminant analysis of principal components (DAPC) to infer group structure for the number of groups K = 3–5 (obtained with the function *find*.*clusters*.) (Table S3) and produced using the R software version 3.5.1. Each genotype is a bin on the x-axis, and the assigned probability of population membership is shown as a stacked bar chart. Each population is shown in different color. Overall for K = 3, group 1: GxM, group 2: WI, group 3: M; for K = 4, group 1: GxWI and MxWI, group 2: GxM, group 3: WI, group 4: M; for K = 5, group 1: GxWI and MxWI, group 2: WI, group 3: G, group 4: GxM, group 5: M.

In order to describe the diversity between pre-defined groups, Discriminant Analysis of Principal Components (DAPC) was performed to obtain the number of clusters. These results were consistent with the cross-validation errors (ADMIXTURE) and Evanno algorithm (STRUCTURE) regarding the number of clusters (K). K = 4 was again revealed as the most likely scenario, closely followed by K = 3 and K = 5 (Fig. 3) (Supplementary Table S2). At K = 3, accessions were divided in agreement with the other methods (ADMIXTURE and STRUCTURE). One group included mainly Guatemalan race accessions and GxM hybrids. A second group consisted of West Indian race accessions, GxWI hybrids and MxWI hybrids. The third group included Mexican race genotypes, GxM hybrids and MxWI hybrids (Supplementary Table S2). For K = 4, the West Indian race accessions were divided into two groups, one which included mainly pure West Indian genotypes and another one which included mainly GxWI hybrid genotypes. For K = 5, Guatemalan genotypes and GxM hybrid genotypes were split into two different groups (Supplementary Table S2).

In order to validate the pre-defined clusters shown above, the fixation index (Fst value) was calculated for every pair of populations using the pre-defined groups (K = 3–5) by DAPC (Supplementary Table S2). In all cases, a contrast between populations was shown and supported the previous analysis. For K = 4, the lowest value was 0.18 between groups two (mostly genotypes considered as GxM hybrids, and some cultivars considered Guatemalan) and one (mostly cultivars considered as GxWI hybrids). The highest value was 0.61 between groups three (mostly cultivars considered as West-Indian) and two (mostly cultivars considered as GxM hybrids) (Table 3).

**Table 3:** Fst genetic differentiation of 71 avocado accessions grouped by K = 4.

	Group1 [GxWI]	Group2 [G] + [GxM]	Group3 [WI]	Group4 [M]
Group1 (GxWI)	0	0.18	0.39	0.23
Group2	0.18	0	0.61	0.33
Group3 (WI)	0.39	0.61	0	0.48
Group4 (M)	0.23	0.33	0.48	0

The most represented race per group is shown inside the parentheses.

Nucleotide diversity was also studied for each cluster using different indexes (Pi and Watterson’s Theta) (Table 4). For K = 4, Pi ranged from 270.14 to 515.27, and Watterson’s Theta ranged from 304.74 to 471.15. A higher diversity was obtained in the cluster with mainly Mexican genotypes, followed by the cluster with mainly West Indian and Guatemalan genotypes, whereas a lower diversity was shown in the group with mainly GxM hybrids.

**Table 4:** Nucleotide diversity statistics according to population structure (K = 3, K = 4, and K = 5) performed by DAPC.

	Groups	Number of accessions	Pi	Watterson’s Theta
K = 3	1 (GxM)	37	273.65	307.58
	2 (WI)	22	543.69	521.76
	3 (M)	12	515.27	471.15
K = 4	1 (GxWI)	14	419.23	467.9
	2 (GxM)	35	270.14	304.74
	3 (WI)	10	417.75	434.08
	4 (M)	12	515.27	471.15
K = 5	1 (GxWI)	12	420.06	458.96
	2 (WI)	10	417.75	434.08
	3 (G)	13	293.23	303.88
	4 (GxM)	24	234.76	264.03
	5 (M)	12	515.27	471.15

The accessions belonging to each group are specified in the Supplementary Table S3.The most represented race per group is shown inside the parentheses.

The genetic diversity per group established by DAPC and minor allele frequencies were also analyzed. The highest observed heterozygosity (0.20) was shown in the cluster with mainly Mexican race cultivars and, in the case of minor allele frequencies, the highest values (0.11) were observed in the same group (Table 5).

**Table 5:** Proportion of observed heterozygosity (Ho) and average minor allele frequency for K = 3, K = 4, and K = 5.

	Groups	Number of accessions	Proportion observed heterozygosity (Ho)	Average Minor allele frequency
K = 3	1(GxM)	37	0.14	0.08
	2(WI)	22	0.15	0.10
	3(M)	12	0.20	0.11
K = 4	1(GxWI)	14	0.19	0.11
	2(GxM)	35	0.14	0.08
	3(WI)	10	0.10	0.07
	4(M)	12	0.2	0.11
K = 5	1(GxWI)	12	0.19	0.11
	2(WI)	10	0.10	0.07
	3(G)	13	0.14	0.10
	4(GxM)	24	0.14	0.10
	5(M)	12	0.20	0.11

The most represented race per group is shown inside the parenthesis.

### Assignment of genotypes of unknown or confusing pedigree to established groups

Based on the above analyses, the assignment of some genotypes of unknown or confusing pedigree to racial groups could be established. Among known genotypes with ambiguous racial assignments, examples include ‘Bacon’, ‘Edranol’, ‘Fuerte’, ‘Gem’, ‘Gwen’, ‘Hass’, ‘Lyon’, ‘Pinkerton’, ‘Toro Canyon’ and ‘TX531’ which have been considered by different authors as pure Mexican^40^, Guatemalan^4,12,41^ or GxM hybrids^4,11,12^ (Table 2). The ADMIXTURE results obtained in this work indicate that all are indeed GxM hybrids, although in ‘Edranol’ a West Indian component was also found. Some samples whose pedigree was unknown (‘A0.25’, ‘A0.68’, ‘87.17.1’, ‘1.14.2’ and ‘Alcaraz’) seem to be GxM hybrids although some probably are three-race hybrids with a low proportion of West Indian heritage. Other accessions (‘Mike’ and ‘Mrs Tooley’) seem to be pure Guatemalan whereas others (‘Hansie’ and ‘C.A. Bueno’) appear as pure Mexican.

## Discussion

Although numerous crop breeding programs are benefiting from new molecular genotyping approaches, these advances are slower in most woody perennial species and especially in tropical and subtropical fruit crops since, in most cases, no previous significant genomic information is available. Regarding avocado, in spite of the different ongoing breeding programs and different types of molecular markers that have been developed and used in the last two decades^5,8,10,14–19,28–31,40,42,43^, there is still a need to generate additional markers that can be used at a large scale, especially to link molecular markers to most of the traits of agronomic interest, that are controlled by multiple genes. Thereby, the use of new approaches such as high throughput sequencing can fill this gap in order to speed up avocado breeding as has occurred in other crops.

### A draft ‘Hass’ avocado genome for diversity analyses

In this study an avocado (cv. ‘Hass’) fragmented genome with small contigs was developed. This fragmentation presents several limitations for genomic studies, such as the impossibility to perform a gene structure annotation, and, consequently, its use for gene discovery. Nevertheless, this draft genome allowed aligning the reads from a reduced-representation approach, and obtaining a high number of molecular markers. Since the use of non-reference variant calling approaches such as Stacks^44^, TASSEL-UNEAK^45^ and GBS-SNP-CROP^46^ can increase the possibilities of variant miscalls^46–48^ the approach followed in this work using a fragmented genome draft is appropriate to reduce this problem. Previous studies have developed some SNP markers in avocado^28–31,43^ but, to our knowledge, this is the first time that an avocado draft genome has been used to facilitate SNP calling from a reduced-representation sequencing. Current work is underway to generate a reference genome of avocado starting from the draft ‘Hass’ genome developed in this work.

### Diversity analyses and population structure

A total of  7,108 Single-Nucleotide Polymorphism (SNPs) were detected for the 71 accessions studied using a ‘Hass’ draft genome to align the reads. These molecular markers showed a higher proportion of transition substitutions (61.10%) over transversions (38.89%). This is commonly known as ‘transitions bias’ and it is explained by the fact that transitions are more conservative on proteins and has been reported in previous studies with different crops including avocado^28,49–51^. Probably due to the lack of sterility barriers between the avocado horticultural races, a low percentage (19.45%) of private SNPs was observed.

The average observed heterozygosity (0.16) was lower than the results reported in other studies based on simple sequence repeat (SSR) markers^15–17^ and with different accessions than those analyzed in this work. These differences have been obtained in other studies^50,52^ and were expected considering the nature of SSRs^49,53^. A lower level of observed heterozygosity was also reported compared to other woody perennial crops such as peach, litchi or olive^54–56^. These differences could be due to the kind of accessions considered. Thus, avocado market worldwide is currently dominated by a single cultivar, ‘Hass’, whereas in other fruit crops, as peach and olive, a wide range of cultivars is grown around the world. ‘Hass’ or ‘Hass’ descendants, such as ‘Gwen’, are part of the pedigree of different varieties in the GxM group (the most representative in this study) and this biased selection could result in a decrease of heterozygosity.

In this work, different analyses utilizing SNP markers (PCA, Neighbour-Joining, ADMIXTURE, STRUCTURE, and DAPC) were performed. These show a clear separation between horticultural races, although with exceptions in some STRUCTURE and DAPC results, in which a clear distinction between genotypes considered as Guatemalan and GxM hybrids was not obtained for K = 4 in contrast to ADMIXTURE with which a separation between those two groups was found. This difficulty in separating both groups was expected since Guatemalan genes predominate in current avocado germplasm^57^. Moreover, as there are not sterility barriers among the botanical races, admixture between different races may have occurred during avocado evolutionary history and domestication processes^2^. In any case, overall, the clustering inferred with DAPC resulted in lower admixture among accessions than that inferred with either STRUCTURE or ADMIXTURE. Similar results of genetic admixture underestimation with DAPC have been shown in other studies and could be due to overestimation of posterior membership probability by DAPC^58,59^. Interestingly at K = 5 a new subgroup is obtained with ADMIXTURE (Fig. 2b) in the GxM group. This new group could represent accessions with a higher Mexican component.

The group with mainly Mexican race accessions shows the highest genetic diversity and the highest proportion of private SNPs (46.42%) (Supplementary Table S3) together with a high observed heterozygosity. Similar results were also obtained in other studies^11,12,16^. Regarding the genetic diversity results, it should be noted that the group with mainly Guatemalan accessions and the group with mainly Mexican accessions show a higher genetic diversity than the GxM hybrid group, despite their lower sample size. The results obtained also show a clear separation of West Indian accessions from the two other horticultural races as has been reported in previous studies^9,16,18,40^ using a lower number of molecular markers. This is expected taking into account that the Mexican and Guatemalan races have a common ecological niche, in the tropical highlands, whereas the West Indian race is adapted to lowlands in Central America^2^.

### Assignment of genotypes of unknown pedigree to established groups

In avocado the main criteria to assign genotypes to the three specific botanical races have been based on morphological traits and, since most of the accessions are developed from chance seedlings, their pedigree is unknown. The approach followed in this work allowed the assignment of some unknown or unclear genotypes to established groups. In agreement with previous works^40^, admixture among the three botanical races are shown for some cultivars, although GxM genotypes involve most of the accessions studied. These hybrids represent the most important avocado cultivars grown worldwide.

In this study, the development of a high number of SNPs after mapping the raw read to a draft avocado (cv. ‘Hass’) genome has allowed the genotyping and efficient discrimination of avocado accessions revealing a clear grouping based on racial origin. The SNP markers developed are a public resource that will be useful for future studies of avocado germplasm management and characterization, Genetic Selection (GS), Marker Assisted Selection (MAS), Genome Wide Association Studies (GWAS) or Quantitative Trait Loci (QTL) analyses and, consequently, helping to significantly reduce breeding costs in this crop. However, this progress will need additional studies to increase the number of available markers in order to have an optimum number of markers in the different avocado breeding populations.

## Methods

### Plant material

Seventy one avocado (*Persea americana* Mill.) accessions were selected and young leaves were collected in the field. The accessions analyzed combine genotypes from the different avocado races obtained from breeding programs (such as ‘Gem’, ‘Gwen’, ‘Iriet’ or ‘Lamb Hass’), commercial varieties (‘Bacon’, ‘Choquette’, ‘Edranol’, ‘Fuerte’, ‘Hass’ or ‘Reed’), rootstocks (‘Dusa’, ‘Thomas’ or ‘Toro Canyon’) and local Spanish accessions with interest as possible source of new rootstocks (‘La Piscina’ or ‘C.A. Bueno’). Those accessions are maintained in three different germplasm collections: IHSM La Mayora (IM; Algarrobo Costa, Spain), Westfalia Fruit (WF; Tzaneen, South Africa) and the US National Avocado Germplasm Repository (UA; Miami, FL, US) (Table 2). Two different samples of ‘Hass’ from two different germplasm collections were included in the analyses as control of the results obtained.

### DNA extraction, library preparation, sequencing and processing the raw reads

DNA from leaves of each accession was isolated using a Qiagen DNeasy Plant Mini Kit following the manufacturer’s guidelines. The DNA purity and concentration were determined using NanoDrop spectrophotometer and Qubit 2.0 Fluorometer. The optimization of a library enzyme was performed on a ‘Hass’ genomic DNA sample digested with PstI, EcoT221, and ApeKI restriction enzymes. The DNA fragment distribution was assessed with Agilent 2100 Bioanalyzer System. Libraries were prepared using Sonah *et al*.^60^ protocol digesting 100 ng genomic DNA of each variety with ApeKI. The resulting libraries were sequenced with the Illumina HiSeq 2500 platform (1 × 100) at the Duke Center for Genomics and Computational Biology and the Illumina HiSeq 4000 platform (2 × 150) at the Novogene Corporation.

The raw reads were demultiplexed using GBSx package^61^. Then reads were processed to remove possible adapter sequences, discard reads shorter than 50 bases and filter low-quality regions by using Fastq-mcf software version 1.04.807^62^ (-l 50 and -q 30).

### A draft avocado (cv.‘Hass’) genome assembly

In order to map the reads to a draft avocado genome, the ‘Hass’ genotype was sequenced (2 × 150) with a depth of 100X using the Illumina platform. The genome size and heterozygosity were estimated using the Kmer distribution approach described in Liu *et al*. 2013^63^. In brief, Kmer distributions for 19, 25, 31, 37, 43, 55, 61, 67, 73 and 85-mers were calculated with Jellyfish and then loaded in the GenomeScope web portal^64^. Two different assemblers were used to assemble the Illumina reads, Minia^65^ and SOAPdenovo2^66^. Although both of them use algorithms for de novo short read assemblies, Minia requires lower computational resources that SOAPdenovo2 and filters false positives^65^. Kmer sizes ranging from 17 to 115-mers (steps of 8) were used with both assemblers. The assembled contigs stats were compared across the different conditions and assemblers and the assembly produced by Minia^65^ with a Kmer of 115 was selected as the one that produced the most contiguous assembly as reported in other studies^65^. Contigs were scaffolded using SSPACE v3.0^67^.

### Mapping, SNP discovery and filtering

The generated reads were mapped with BWA version 0.7.10-r789^68^ with default parameters. Unmapped reads were removed using Samtools version 1.3.1^69^ and BAM files were produced with the retained reads. All BAM files were merged by Bamaddrg (https://github.com/ekg/bamaddrg), and Samtools package version 1.3.1^69^ was used to sort and index BAM files. FreeBayes version 0.9.20^70^ was run to detect variants and remove SNPs with mapping quality lower <20 and read depth <5. The raw SNPs obtained were further filtered using the VCFtools package version 0.1.12.^71^ removing no biallelic SNPs, missing data and SNPs within 1000 bp distance. Before and after filtering, a summary statistic was generated using Vcf-stats version 0.1.12^71^. Finally, only SNP variants were retained and their diversity was analyzed using Adegenet package version 2.1.1^72^ and Hardy-Weinberg equilibrium was tested using pegas package version 0.10^73^.

### Analysis of the genetic structure of diverse avocado accessions

In order to show the usefulness of the SNPs generated, the genetic relationships, genetic structure and group divergence of 71 avocado accessions were thoroughly analyzed using different methods such as PCA, NJ distance tree, DAPC and Bayesian clustering as well as genetic properties of these populations through parameter such as Fst, Pi and Watterson’s theta.

PCA was performed using Adegenet package version 2.1.1^72^ and was plotted using ggplot2 packages version 3^74^ in RStudio version 1.1.453^75^ and R version 3.5.1.

Prevosti’s distance ($D\,{\Pr }evosti\,(a,b)=\,\frac{1}{2r}\,\mathop{\sum }\limits_{k=1}^{\upsilon }\,\mathop{\sum }\limits_{j=1}^{m(k)}\,|Pajk-Pbjk|$ where $\upsilon$ is the number of loci considered, *Pajk* the frequency of the allele arrangement k in the locus j in the population a, and *Pajk* the corresponding value in the population b^36^) matrix and Neighbor-joining (NJ) tree were generated via the Poppr package version 2.8.2^76,77^ with 2000 bootstrap replicates using the SNP data set. The figures were plotted with FigTree version 1.4.4^78^.

The population structure was studied with three different approaches (ADMIXTURE, STRUCTURE and DAPC). The three programs basically assign each of the accessions to one or more ancestral populations or clusters. They differ in how the data are processed and the algorithm used. Thus, maximum likelihood estimation of individual ancestries was analyzed with ADMIXTURE version 1.3^37^ that was run iterating K from 1 to 20. This analysis is based on the same statistical model as STRUCTURE although it performs a maximum likelihood estimation of individuals instead of a Bayesian approach and, consequently, allows a faster cluster estimation from a large SNP dataset. Furthermore, in order to choose the optimum number of populations (K), a cross-validation approach was used for all the Single Nucleotide Polymorphism (SNPs). Each chosen value of K was plotted using RStudio version 1.1.453^75^ and R version 3.5.1. The STRUCTURE program was run five times per each number of populations (K). Each run was implemented with a burn-in period of 20000 steps followed by 200000 Monte Carlo Markov chain replicates^79–81^ Evanno *et al*.^82^ method was used to determine the most probable number of K with the software STRUCTURE HARVESTER^39^. Subsequently, since STRUCTURE-like approaches assume that markers are not linked and that populations are panmictic^38^, Discriminant Analysis of Principal Components (DAPC) was also applied in order to identify and describe well-defined clusters of genetically related genotypes using the R package Adegenet version 2.1.1^72^. To perform this analysis, data were transformed using PCA. The find.clusters function was used to identify the number of clusters. The Bayesian Information Criterion (BIC) was calculated to associate with the correct number of subgroups, and a cross-validation function (XvalDapc) was used to corroborate the best number of PCA retained. Before this analysis, the files were read using read.vcf and converted into Genind and Genlight class with VcfR2genind and VcfR2genlight.

Finally, the Fixation index (Fst) which allows differentiating populations with ranges between 0 (no differentiation) and 1 (complete differentiation)^83^ was also obtained with the R package PopGenome version 2.6.1^84^ to analyze group distinction. Moreover, Nucleotide diversity statistics Pi and Watterson’s theta were estimated considering the grouping produced by DAPC, K = 3, K = 4, and K = 5 and were also determined with the same package.

## Supplementary information

Supplementary materials

## Data Availability

The ‘Hass’ draft genome raw reads have been deposited at NCBI under the BioProject PRJNA564097. The GBS dataset is deposited under PRJNA564105. Most of the analyses have been carried out using R software 3.5.1. All scripts have been deposited at https://github.com/IHSMFruitCrops/Hass-genotyping.
