# Author Correction: Genome-Wide SNP discovery and genomic characterization in avocado (*Persea americana* Mill.)

**DOI:** 10.1038/s41598-023-29346-w

**Published:** 2023-02-21

**Authors:** Alicia Talavera, Aboozar Soorni, Aureliano Bombarely, Antonio J. Matas, Jose I. Hormaza

**Affiliations:** 1 Instituto de Hortofruticultura Subtropical y Mediterránea La Mayora (IHSM La Mayora -UMA-CSIC), Algarrobo-Costa, 29751 Málaga, Spain; 2 Department of Biotechnology, College of Agriculture, Isfahan University of Technology, Isfahan, 84156-83111 Iran; 3 School of Plant and Environmental Sciences, Virginia Tech, Blacksburg, VA USA; 4 Department of Biosciences, Università degli Studi di Milano, Milan, Italy; 5 Departamento de Biología Vegetal, Universidad de Málaga, Málaga, Spain

Correction to: *Scientific Reports* 10.1038/s41598-019-56526-4, published online 27 December 2019

This Article contains an error in Affiliation 2, which is incorrectly given as ‘Department of Biotechnology, College of Agriculture, University of Technology, Isfahan, 84156-83111, Iran’. The correct affiliation is listed below:

Department of Biotechnology, College of Agriculture, Isfahan University of Technology, Isfahan, 84156-83111, Iran

