# Supplementary material for: Genome-Wide SNP discovery and genomic characterization in avocado (Persea americana Mill.)
Source: Sci Rep. 2019 Dec 27;9:20137. doi: 10.1038/s41598-019-56526-4 (PMC6934854; doi:10.1038/s41598-019-56526-4)
Supplement: Supplementary file 1 — Supplementary materials [file 41598_2019_56526_MOESM1_ESM.pdf]

**Supplementary Information**

Genome-Wide SNP discovery and genomic characterization in avocado (*Persea americana* Mill.).

Alicia Talavera[1], Aboozar Soorni[2], Aureliano Bombarely[3,4], Antonio J. Matas[1,5] & Jose Iñaki Hormaza[1] *

[1]Instituto de Hortofruticultura Subtropical y Mediterránea La Mayora (IHSM-UMA-CSIC), Algarrobo-Costa, Málaga, Spain.

[2]Department of Biotechnology, College of Agriculture, University of Technology, Isfahan 84156-83111, Iran.

[3]School of Plant and Environmental Sciences, Virginia Tech, Blacksburg, VA. USA.

[4]Department of Biosciences Università degli Studi di Milano, Milan, Italy.

[5]Departamento de Biología Vegetal, Universidad de Málaga, Spain.

**Corresponding author**

ihormaza@eelm.csic.es

**Supplementary Table S1**. Private SNPs in the 71 avocado cultivars analyzed in this work.

| SampleID | Private SNPs |
|---|---|
| 2835 | 6 |
| A06852 | 1 |
| 871728 | 1 |
| BACO39 | 17 |
| BETA19 | 5 |
| BUTL16 | 5 |
| CABU95 | 41 |
| CATA11 | 6 |
| CHOQ9 | 7 |
| COLL1 | 6 |
| COLI31 | 2 |
| COLL36 | 75 |
| DUSA33 | 21 |
| FUCH17 | 6 |
| G692 | 2 |
| GEM77 | 1 |
| GOTT04 | 41 |
| H28757 | 104 |
| HANS05 | 246 |
| HASS38 | 1 |
| IRIE34 | 8 |
| A06729 | 8 |
| LAHA24 | 1 |
| LAPI93 | 13 |
| LARG24 | 338 |
| LIND50 | 9 |
| LISA23 | 12 |
| MELE12 | 4 |
| MIKE30 | 4 |
| MONR10 | 4 |
| MRTO08 | 28 |
| MUGR27 | 8 |

| | |
|---|---|
| NABA21 | 18 |
| NECR31 | 81 |
| NIML09 | 1 |
| POLL6 | 3 |
| REED89 | 8 |
| RURO36 | 21 |
| RUSS22 | 10 |
| RYAN13 | 11 |
| SEMI14 | 3 |
| TEAG60 | 11 |
| TELE66 | 37 |
| THOM90 | 42 |
| TOCA96 | 64 |
| TRAP2 | 1 |
| TX5344 | 2 |
| VERO4 | 28 |
| WALD28 | 1 |
| WEST5 | 8 |
| YON3 | 2 |
| TOTAL | 1383 |

**Supplementary Table S2**. Classification of each sample per cluster (K=2-4) performed by DAPC.

| K=3 | | K=4 | | K=5 | |
|---|---|---|---|---|---|
| GROUP 1 | COLI31 | GROUP 1 | YON3 | GROUP 1 | YON3 |
| | HASS38 | | CHOQ9 | | CHOQ9 |
| | REGA11 | | MONR10 | | MONR10 |
| | MALU85 | | MELE12 | | MELE12 |
| | RINC12 | | SEMI14 | | SEMI14 |
| | RYAN13 | | FUCH17 | | FUCH17 |
| | EDRA63 | | LISA23 | | LISA23 |
| | GWEN40 | | LARG24 | | LARG24 |
| | REED89 | | COLL36 | | COLL36 |
| | FUER16 | | 114218 | | RR8691 |
| | MRTO08 | | RR8691 | | G692 |
| | SHEP42 | | G692 | | RURO36 |
| | 114218 | | RURO36 | GROUP 2 | COLL1 |
| | NN1068 | | ALCA74 | | TRAP2 |
| | TX5344 | GROUP 2 | COLI31 | | WEST5 |
| | A05720 | | HASS38 | | POLL6 |
| | PINK45 | | REGA11 | | CATA11 |
| | NABA21 | | MALU85 | | BUTL16 |
| | CILF46 | | RINT12 | | BERN18 |
| | LAHA24 | | RYAN13 | | BETA19 |
| | NIML09 | | EDRA63 | | RUSS22 |
| | LYON25 | | GWEN40 | | WALD2 |
| | ALCA74 | | REED89 | GROUP 3 | MALU85 |
| | GRAC26 | | FUER16 | | REED89 |
| | LIND50 | | MRTO08 | | MRTO08 |
| | MUGR27 | | SHEP42 | | 114218 |

| | | | | | |
|---|---|---|---|---|---|
| | NN6310 | | NN1068 | | NN1068 |
| | 871728 | | TX5344 | | NABA21 |
| | A06852 | | A05720 | | NIML09 |
| | GEM77 | | PINK45 | | ALCA74 |
| | A06729 | | NABA21 | | LIND50 |
| | MIKE30 | | CILF46 | | MUGR27 |
| | A02554 | | LAHA24 | | NN6310 |
| | H28757 | | NIML09 | | A06852 |
| | HASS55 | | LYON25 | | MIKE30 |
| | IRIE34 | | GRAC26 | GROUP 4 | COLI31 |
| | 2835 | | LIND50 | | HASS38 |
| GROUP 2 | COLL1 | | MUGR27 | | REGA11 |
| | TRAP2 | | NN6310 | | RINT12 |
| | YON3 | | 871728 | | RYAN13 |
| | WEST5 | | A06852 | | EDRA63 |
| | POLL6 | | GEM77 | | GWEN40 |
| | CHOQ9 | | A06729 | | FUER16 |
| | MONR10 | | MIKE30 | | SHEP42 |
| | CATA11 | | A02554 | | TX5344 |
| | MELE12 | | H28757 | | A05720 |
| | SEMI14 | | HASS55 | | PINK45 |
| | BUTL16 | | IRIE34 | | CILF46 |
| | FUCH17 | | 2835 | | LAHA24 |
| | BERN18 | GROUP 3 | COLL1 | | LYON25 |
| | BETA19 | | TRAP2 | | GRAC26 |
| | RUSS22 | | WEST5 | | 871728 |
| | LISA23 | | POLL6 | | GEM77 |
| | LARG24 | | CATA11 | | A06729 |
| | WALD28 | | BUTL16 | | A02554 |
| | COLL36 | | BERN18 | | H28757 |
| | RR8691 | | BETA19 | | HASS55 |

| | | | | | |
|---|---|---|---|---|---|
| | G692 | | RUSS22 | | IRIE34 |
| | RURO36 | | WALD28 | | 2835 |
| GROUP 3 | VERO4 | GROUP 4 | VERO4 | GROUP 5 | VERO4 |
| | DUSA33 | | DUSA33 | | DUSA33 |
| | BACO39 | | BACO39 | | BACO39 |
| | TELE66 | | TELE66 | | TELE66 |
| | THOM90 | | THOM90 | | THOM90 |
| | LAPI93 | | LAPI93 | | LAPI93 |
| | CABU95 | | CABU95 | | CABU95 |
| | TOCA96 | | TOCA96 | | TOCA96 |
| | GOTT04 | | GOTT04 | | GOTT04 |
| | NECR31 | | NECR31 | | NECR31 |
| | HANS05 | | HANS05 | | HANS05 |
| | TEAG60 | | TEAG60 | | TEAG60 |

**Supplementary Table S3**. Private SNPs segregating in the groups stablished by DAPC. Percentages are of 7108 private SNPs. The most represented race per group is shown inside the parenthesis.

| | Groups | Private SNPs | SNPs % |
|---|---|---|---|
| **K=3** | 1 (GxM) | 222 | 16.05 |
| | 2 (WI) | 519 | 37.52 |
| | 3 (M) | 642 | 46.42 |
| **K=4** | 1 (GxWI) | 474 | 34.27 |
| | 2 (GxM) | 222 | 16.05 |
| | 3 (WI) | 45 | 3.25 |
| | 4 (M) | 642 | 46.42 |
| **K=5** | 1 (GxWI) | 474 | 34.27 |
| | 2 (WI) | 45 | 3.25 |
| | 3 (G) | 77 | 5.56 |
| | 4 (GxM) | 145 | 10.48 |
| | 5 (M) | 642 | 46.42 |

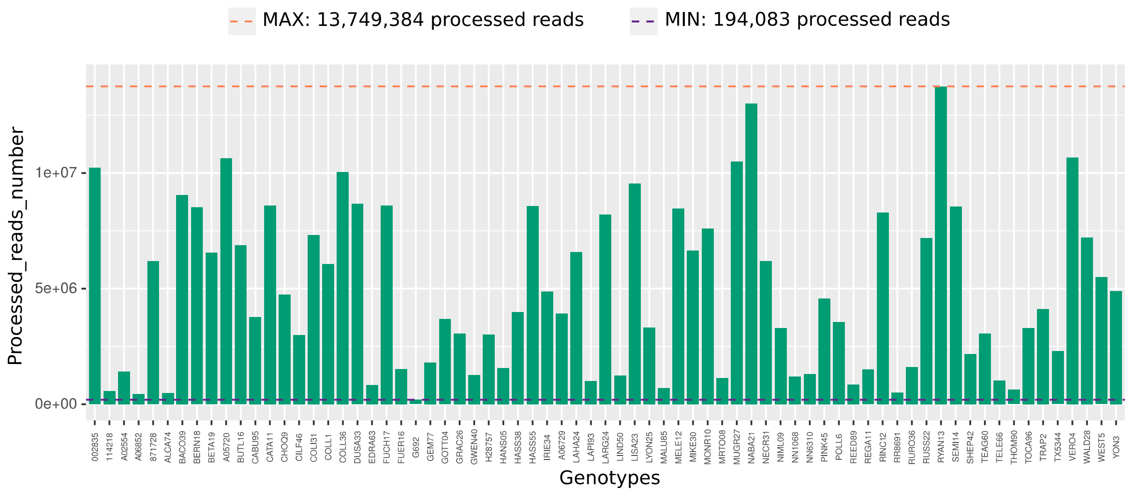


**Figure S1** Processed read number per cultivar. The blue and red lines represent the maximum and minimum number of processed reads, respectively. The barplot was produced using the R software version 3.5.1 with the package ggplot2 version 3[74].

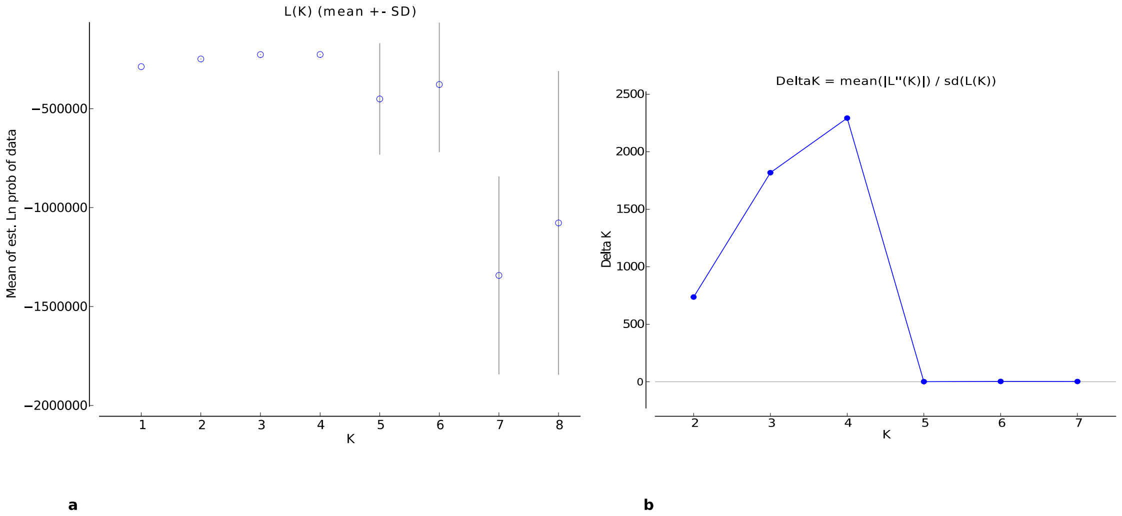


**Figure S2** (a). Data probability for K 1-8. (b). Estimation of the number of groups using the method proposed by Evanno *et al.* (2005). These graphs were produced using STRUCTURE HARVESTER program[40].

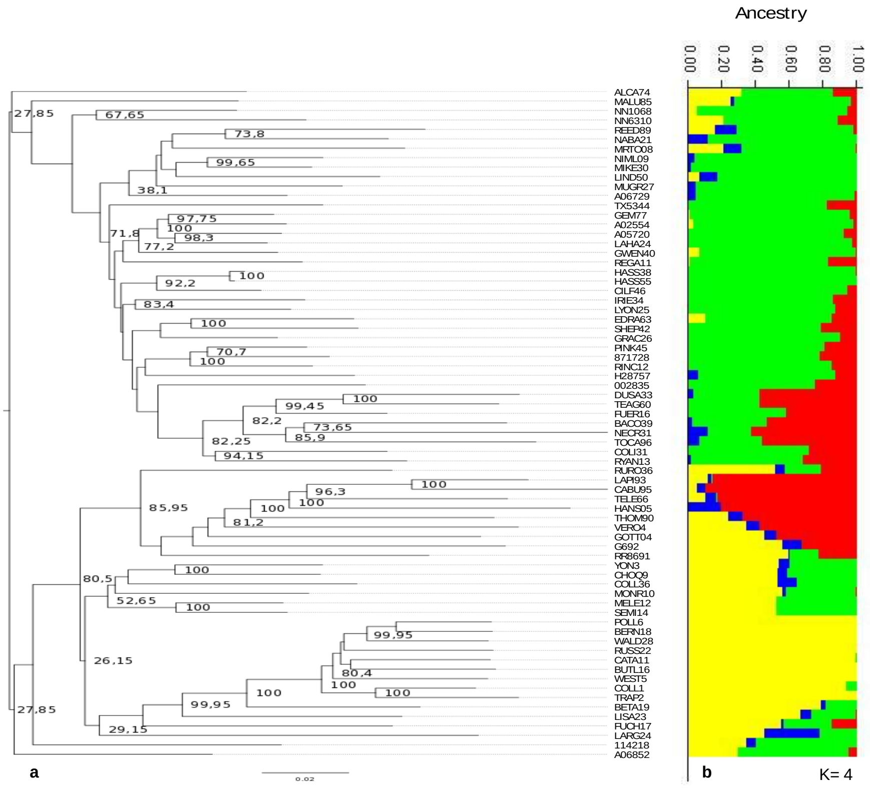


**Figure S3** (a). Dendrogram based on Neighbour Joining (NJ) showing the similarity relationships among 71 avocado accessions. Node labels represent bootstrap values (only values cited in the manuscript and values > 70% are shown) out of 2000 bootstrap replicates. (b). Barplot describing the population stratification of the most informative K values (K=4). Genomic admixture was estimated by STRUCTURE software[39].
